# Mechanism study of lncRNA RMRP regulating esophageal squamous cell carcinoma through miR-580-3p/ATP13A3 axis

**DOI:** 10.1007/s12672-024-00990-6

**Published:** 2024-05-09

**Authors:** ZiRui Tan, ShengJie Luan, XiaoPeng Wang, WenPeng Jiao, Pu Jiang

**Affiliations:** 1 The Fourth Hospital of Hebei Medical University, No. 12, Jiankang Road, Chang’an District, Shijiazhuang City, 050000 Hebei Province China; 2 Department of Tumor Chemoradiotherapy, Central Hospital of Qinghe County, Xingtai City, 054800 Hebei Province China

**Keywords:** lncRNA RMRP, Esophageal squamous cell carcinoma, miR-580-3p, ATP13A3, Glycolysis

## Abstract

**Objective:**

It is well-known that lncRNAs regulate energy metabolism in tumors. This study focused on the action of RMRP on esophageal squamous cell carcinoma (ESCC) cell proliferation, apoptosis, and glycolysis.

**Methods:**

In the resected ESCC tissues and adjacent tissues from patients, RMRP/miR-580-3p/ATP13A3 expressions were evaluated. ESCC cell proliferation rates and apoptotic rates were measured by CCK-8 and flow cytometry, respectively. Apoptosis related markers were examined by Western blot. Moreover, glucose uptake, lactic acid, and ATP were measured by commercial kits, whereas HK2 and PKM2 were evaluated by Western blot to study ESCC cell glycolysis. Finally, the editing program of RMRP/miR-580-3p/ATP13A3 was translated by luciferase reporter assay and RIP analysis.

**Results:**

RMRP and ATP13A3 were induced, while miR-580-3p was reduced in their expression in ESCC tissues. Silencing RMRP reduced proliferation, glycolysis, and anti-apoptosis ability of ESCC cells. RMRP sequestered miR-580-3p to target ATP13A3. Silenced ATP13A3 or overexpressed miR-580-3p rescued overexpressed RMRP-mediated promotion of proliferation, glycolysis, and anti-apoptosis of ESCC cells.

**Conclusion:**

RMRP accelerates ESCC progression through the miR-580-3p/ATP13A3 axis, renewing a reference for lncRNA-based therapies for tumors.

**Supplementary Information:**

The online version contains supplementary material available at 10.1007/s12672-024-00990-6.

## Introduction

Esophageal squamous cell carcinoma (ESCC) is the most common histological subtype of esophageal carcinoma, and its etiology is related to diet, genetic factors, microorganisms, and other environmental factors [1]. Multidisciplinary evaluation and treatment have shown survival benefits, such as endoscopic resection for early ESCC, minimally invasive esophagectomy as primary therapy or post-induction chemoradiotherapy, and immunotherapy for metastatic or persistent ESCC cases [2]. Increased glycolysis is a characteristic of cancer metabolism and glycolysis is a carcinogenic event [3]. Glycolysis is less effective than oxidative phosphorylation in terms of production of adenosine triphosphate (ATP), but cancer cells adapt to this disadvantage by increasing glucose absorption, which in turn increases glycolysis rates [4]. Glycolysis not only supports the metabolic needs of tumor cells, but also provides an immune protective ecological niche to activate malignant proliferation, maintenance, and progression [5]. Considering the contribution of glycolysis in the tumor microenvironment, targeting glycolysis may be a potential target for therapy [6].

Changes in lncRNA expression and its mutation promote tumor development and metastasis [7]. LncRNAs regulate energy metabolism in tumors. Insight into lncRNA-mediated metabolic reprogramming can help identify cellular vulnerabilities to improve tumor diagnosis and treatment [8]. Many lncRNAs [9–11] have been reported to correlate to malignant properties of ESCC cells, including but not limited to glycolysis. Notably, a recent paper has emphasized the clinical association between lncRNA RNA component of mitochondrial RNA processing endoribonuclease (RMRP) and ESCC patients’ poor prognosis and tumor stage [12]. Not only in ESCC, but RMRP dysregulation has also been addressed in other cancers and is commonly considered a promoter in tumor malignancy [13–15]. Nevertheless, more understandings of RMRP-related mechanisms are still lacking in ESCC.

miR-580-3p has been identified to be sponged by RMRP [16]. In addition to that, bioinformatics analysis also demonstrated the binding possibility of miR-580-3p and RMRP. In fact, miR-580-3p could be either a tumor inhibitor [17] or a tumor inducer [18] depending on the tumor type. However, it specific property in ESCC lacks scientific investigations until now. Therefore, miR-580-3p, as a downstream molecule of RMRP, was studied in ESCC.

The current research planned to unravel RMRP-mediated proliferation, apoptosis, and glycolysis processes in ESCC and further determined its interplay with miR-580-3p and the downstream target ATP13A3, hoping to provide more evidence for molecule-based targeted therapies for ESCC.

## Materials and methods

### Clinical samples

From April 2017 to March 2021, a total of 53 pairs of ESCC tissues and paracancer normal tissues were gained from The Fourth Hospital of Hebei Medical University. All tissues after identification by two independent pathologists were frozen in liquid nitrogen and stored at − 80 °C. All patients treated with radiation, chemotherapy, or other drugs were removed. The study was approved by the Ethics Committee of The Fourth Hospital of Hebei Medical University (No. 20206HB10). The patient had signed informed consent.

### Cell culture

ESCC lines (KYSE150, TE-1, TE-10, EC-1, and EC-109) and normal esophageal epithelial cells (NE1) were purchased from National Collection of Authenticated Cell Cultures (Shanghai, China). All cells were cultured in Dulbecco’s modified Eagle medium DMEM (Gibco, USA) or RPMI1640 media (Gibco) supplementary with 10% fetal bovine serum (FBS, Gibco), 100 U/ml penicillin and 100 μg/ml streptomycinn (both from SigmaAldrich, St. Louis, MO, USA)., with a humid incubator at 37 °C and 5% CO_2_.

### Nuclear-cytoplasmic fractionation and quantitative PCR

Cytoplasmic and nuclear RNA were isolated by PARIS™ Kit (Invitrogen). Trizol reagent (Invitrogen) was employed to gain total RNA which was then processed with reverse transcription to obtain cDNA of lncRNA and mRNA using PrimeScriptTMII First Strand cDNA Synthesis Kit (Takara, Japan) and that of miRNA using miScript reverse transcription kit (Qiagen, USA). Quantitative PCR was completed using Power SYBR_green PCR master mix (Applied Biosystems) in the ABI 7500 PCR machine. GAPDH and U6 serve as endogenous reference genes. 2^−ΔΔCt^ was adopted to analyze gene levels. Table 1 exhibits primer sequences used for PCR.

**Table 1:** Primers for PCR

Genes	Primers (5′–3′)
LncRMRP	Forward: 5′-GAGGACTCTGTTCCTCCCCT-3′
	Reverse: 5′-TACGCTTCTTGGCGGACTTT-3′
miR-580-3p	Forward: 5′-GCGCTTGAGAATGATGAATC-3′
	Reverse: 5′-TGGTGTCGTGGAGTCG-3′
ATP13A3	Forward: 5′-TGTGGCACAAAGACCACCTT-3′
	Reverse: 5′-CCAAAACCCGCTTCCTGTTG-3′
U6	Forward: 5'- CTCGCTTCGGCAGCACA-3′
	Reverse: 5′-AACGCTTCACGAATTTGCGT-3′
GAPDH	Forward: 5′-CACCCACTCCTCCACCTTTG-3′
	Reverse: 5′-CCACCACCCTGTTGCTGTAG-3′

*LncRMRP* long noncoding RNA RNA component of mitochondrial RNA-processing endoribonuclease, *miR-580-3p* microRNA-580-3p, *GAPDH* glyceraldehyde-3-phosphate dehydrogenase

### Fluorescence in situ hybridization

Fluorescence In Situ Hybridization (FISH) assay was conducted using the FISH Kit (RiboBio, China) according to the manufacturer’s instruction. Briefly, cells were first cultured in 24-well plates for 24 h and then hybridized with 20 μM Cy3-labeled U6, 18S and RMRP. Finally, the images were observed with the fluorescence microscope (Leica, Wetzlar, Germany).

### Northern blot

Northern blot analysis was performed according to a previously published method [32]. DIG Northern Starter Kit (Roche) was used according to the manufacturer’s instruction. The RNA was electrophoresed on a denaturing 7 M urea-6% polyacrylamide gel and subsequently transferred onto Nylon membranes (Roche) for 1 h. Following UV cross-linking, the probes were treated on the membranes and allowed to hybridize overnight. AP reaction was carried out for more than 30 min with antidigoxigenin-AP antibody. Signals were visualized with an ImageQuant LAS 4000 system (GE Healthcare, Charles Cofn, NY, USA).

### Cell transfection

siRNA targeting RMRP and ATP13A3, pcDNA 3.1 overexpression vector, miR-580-3p mimic, miR-580-3p inhibitor and their negative controls were provided by GenePharma (Shanghai, China). KYSE150 cells at 70–80% confluence were conditioned to transient transfection based on Lipofectamine 3000 (Thermo Fisher Scientific) and were harvested after 48 h to evaluate the transfection efficacy by quantitative PCR and Western blot.

### CCK-8

KYSE150 cells (2 × 10^3^) were plated in each well of 96-well plates, in which 10 μL CCK-8 reagent (Jinan Beiaotai Biotechnology Co., Ltd., China) was added at an indicated timepoint, and the absorbance was determined in a microplate reader (Thermo Scientific, Waltham, MA, USA) at 450 nm.

### Flow cytometry

KYSE150 cells were configured into a cell suspension using 100 μL buffer solution and stained with FITC Annexin V and propidium iodide for 10 min avoiding light exposure. Another 400 μL binding buffer was then added before assessing apoptosis by flow cytometry (BD Bioscience, USA).

### Glycolysis measurements

The Glucose Absorption Test Kit (Biovision, CA, USA) measures glucose absorption. The d-lactic acid detection kit and ATP colorimetric/fluorescent detection kit (Biovision) measured the production of lactic acid and ATP, respectively. The corresponding absorbance was detected using a microplate reader.

### Western blot

RIPA lysis buffer (R0010, Solarbio, China) was adopted to harvest total protein of tissues or cells. Afterward, protein concentration was measured with a BCA kit (Yeasen, Shanghai, China) before electrophoresis separation based on sodium dodecyl sulfate–polyacrylamide gel. Then, the treated protein was loaded onto a polyvinylidene fluoride membrane which was then combined with the primary antibody at 4 ℃ overnight and with the diluted HRP-labeled secondary antibody (1:20,000, Abcam) for 1 h. Finally, enhanced chemiluminescence-developed protein signals were quantified using Image J analysis software. Primary antibodies: cleaved Caspase-3 (9664S, Cell Signaling Technology), Bcl‑2 (15071S, Cell Signaling Technology), HK2 (22029-1-AP, Proteintech), PKM2 (4053, Cell Signaling Technology), and GAPDH (ab8245, Abcam).

### Analysis of luciferase activity

Wild-type (WT) RMRP and ATP13A3 3′-UTR fragments containing predicted miR-580-3p binding sites were amplified and inserted into pmirGLO vectors (Promega, USA) to establish the reporters RMRP-WT and ATP13A3-WT. GeneArt™ site-directed mutagenesis PLUS system (A14604; Thermo Fisher Scientific) mutated the putative binding site of miR-580-3p in RMRP and ATP13A3 3' -UTR. The mutant (Mut) RMRP and ATP13A3 3′-UTR were inserted into the pmirGLO vector to construct the reporters RMRP-mut and ATP13A3-mut. KYSE150 cells were co-treated with the reporters and miR-580-3p mimic or mimic NC using Lipofectamine 3000 reagent and collected after 48 h to measure luciferase activity in a dual luciferase reporting system (Promega, Madison, WI, USA) as per protocol.

### RIP experiment

RIP testing was guided under the instruction of RIP kit (Millipore, USA). KYSE150 cells were lysed using a RIP lysis buffer and combined with RIP buffers containing magnetic beads conjugated with human anti-AgO2. Protease K was then applied to digest the protein and isolate the immunoprecipitated RNA. RNA expression was detected by quantitative PCR.

### Data analysis

At least 3 biological replicates were needed for each experiment. All statistical analyses were performed using GraphPad Prism 9.0 software. The student t test analyzed two-group differences, while one-way analysis of variance analyzed multiple-group differences. *P < 0.05 was considered a significant difference.

## Results

### RMRP expression abundance in ESCC

**Fig. 1:**
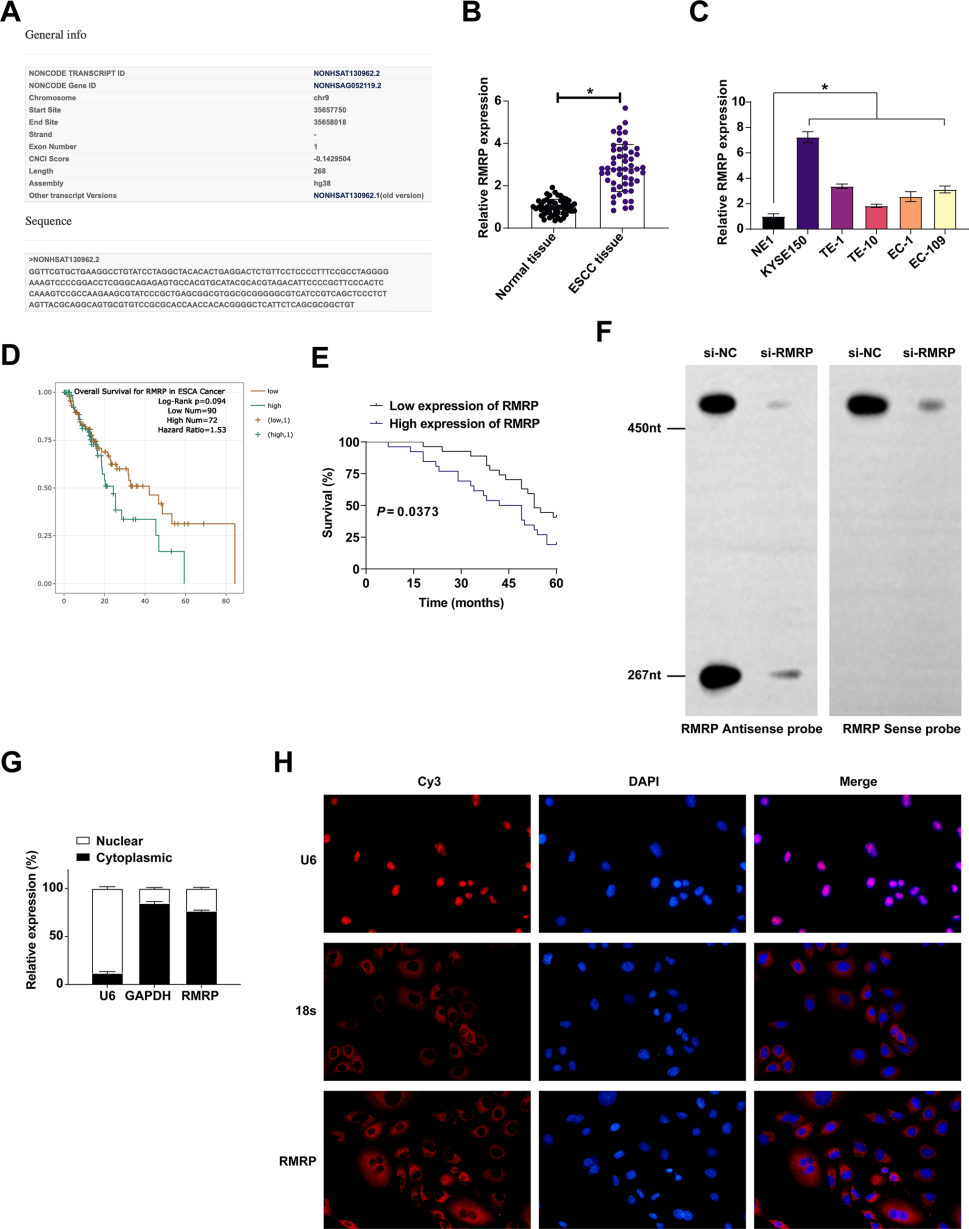
Abnormally high expression of RMRP in ESCC. **A** Bioinformatics website http://www.noncode.org for RMRP gene information. **B** Quantitative PCR assessed RMRP in patients’ tissues. **C** Quantitative PCR assessed RMRP in ESCC cell lines and normal esophageal epithelial cells. **D** Relationship between survival rate and RMRP expression in esophageal carcinoma patients analysed by ENCORI website. **E** Relationship between survival rate and RMRP expression in ESCC patients. **F** Northern blot analysis of RMRP expression in KYSE150 cells. **G** Quantitative PCR assessed RMRP after the fractionation of nuclear and cytoplasmic RNA of KYSE150 cells. **H** Representative RNA-FISH images showing the subcellular location of RMRP in KYSE150 cells, 18S and U6 were used as cytoplasmic and nuclear markers, respectively (Scale bar, 100 μm). Data were expressed as mean ± SD (N = 3). * *P* < 0.05

On the bioinformatics website http://www.noncode.org, RMRP was located at chromosome chr9: 35657750-35658018 [-], with a length of 268 bp (Fig. 1A). Quantitative PCR evaluated RMRP expression signature in ESCC and revealed that RMRP in ESCC tissues and cell lines was higher than that in the normal control (Fig. 1B, C). Chi-square test was conducted to evaluate the correlation between the clinical features of ESCC patients with RMRP expression signature. Table 2 shows that RMRP was highly correlated with tumor size and distal metastasis in ESCC patients. In addition, ENCORI website https://rnasysu.com/encori/index.php analysis revealed patients with high RMRP expression possessed poorer survival than patients with low RMRP expression in esophageal carcinoma which was further confirmed by our finding that patients with high RMRP showed worse 5-year survival in ESCC (Fig. 1D, E).

**Table 2:** Correlation analysis of clinicopathological features in patients with RMRP and ESCC

Characteristic	Cases	The expression of RMRP		P
	n = 53	High (n = 26)	Low (n = 27)	
Age (year)				
≥ 60	41	19	22	0.4649
< 60	12	7	5	
Gender				
Male	30	13	17	0.3412
Female	23	13	10	
Tumor size (cm)				0.0001
≥ 3	19	16	3	
< 3	34	10	24	
TNM stage				
I–II	38	17	21	0.3167
II–IV	15	9	6	
Lymph node metastasis				
Negative	32	9	23	0.0002
Positive	21	17	4	

To further elucidate the mechanisms of RMRP, northern blot analysis of RMRP in KYSE150 cells was performed to confirm that RMRP is a non-coding RNA (Fig. 1F). Nuclear-cytoplasmic fractionation assay and FISH assay were conducted to show that RMRP was mainly located in the cytoplasm of KYSE150 cells (Fig. 1G, H).

### RMRP deficiency inhibits ESCC proliferation and glycolysis and promotes apoptosis

**Fig. 2:**
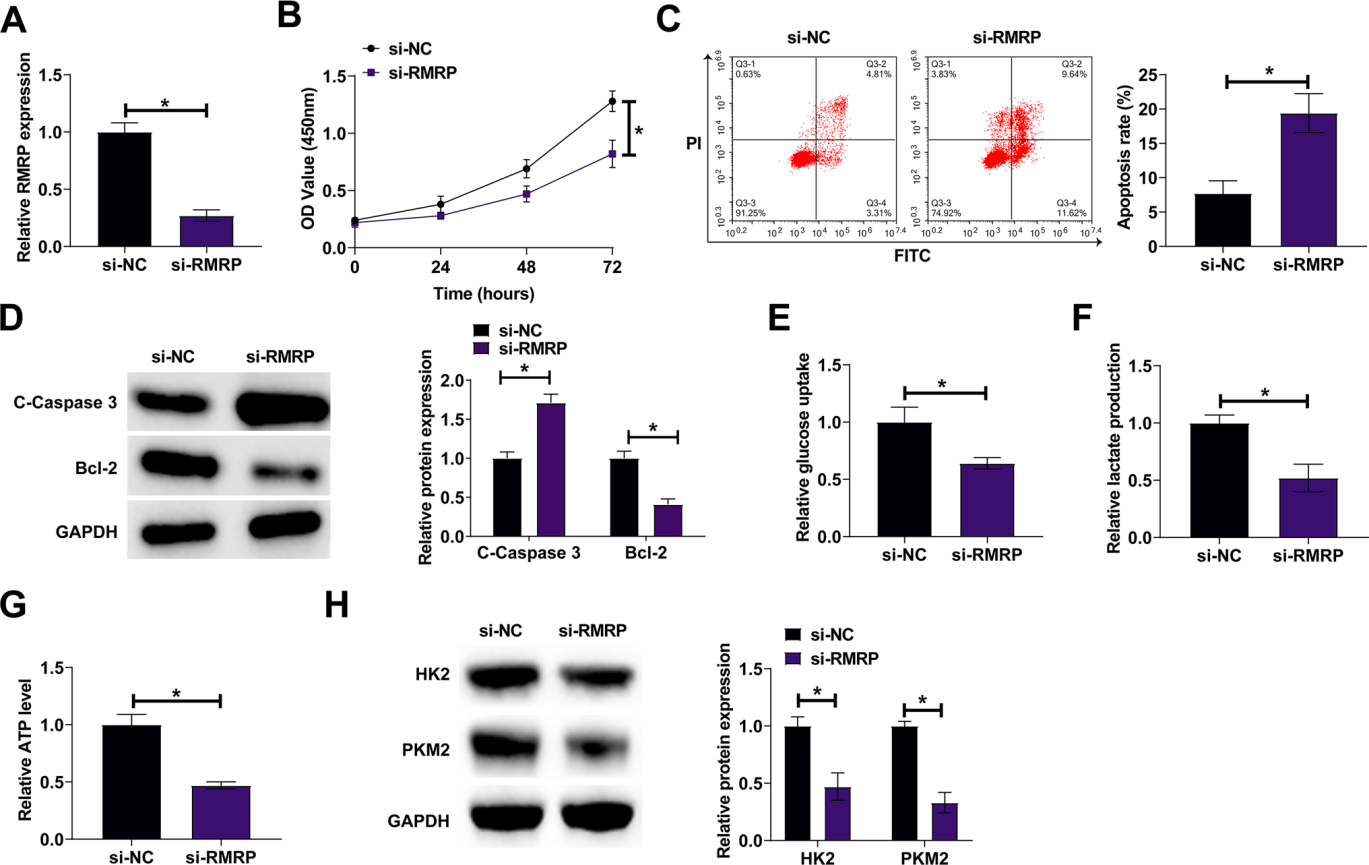
RMRP deficiency inhibits ESCC proliferation and glycolysis and promotes apoptosis. RMRP siRNA was transfected into KYSE150 cells. **A** Quantitative PCR assessed RMRP. **B** CCK-8 assay measured cell proliferation rate. **C** Flow cytometry detected cell apoptosis rate. **D** Western blot evaluated cleaved Caspase-3 and Bcl-2. **E**–**G** Commercial kits detected cellular glucose consumption, lactate production, and ATP levels. **H** Western blot evaluated HK2 and PKM2. Data were expressed as mean ± SD (N = 3). *P < 0.05

siRNA of RMRP was treated in KYSE150 cells, leading to the inhibition of RMRP expression in cells (Fig. 2A). CCK-8 assay showed that inhibiting RMRP reduced cell proliferation rate (Fig. 2B), whereas flow cytometry determined its promoting impact on apoptosis rate (Fig. 2C). Moreover, RMRP silenced ESCC cells showed significantly higher expression of pro-apoptotic protein (cleaved Caspase-3) and significantly lower expression of apoptosis inhibitory protein(Bcl‑2) (Fig. 2D). Changes in cellular glycolysis were then assessed. Knocking down RMRP reduced glucose consumption, lactic acid production, and ATP levels in cells (Fig. 2E–G) and inhibited protein expressions of glycolytic proteins HK2 and PKM2 (Fig. 2H).

### RMRP targets miR-580-3p

**Fig. 3:**
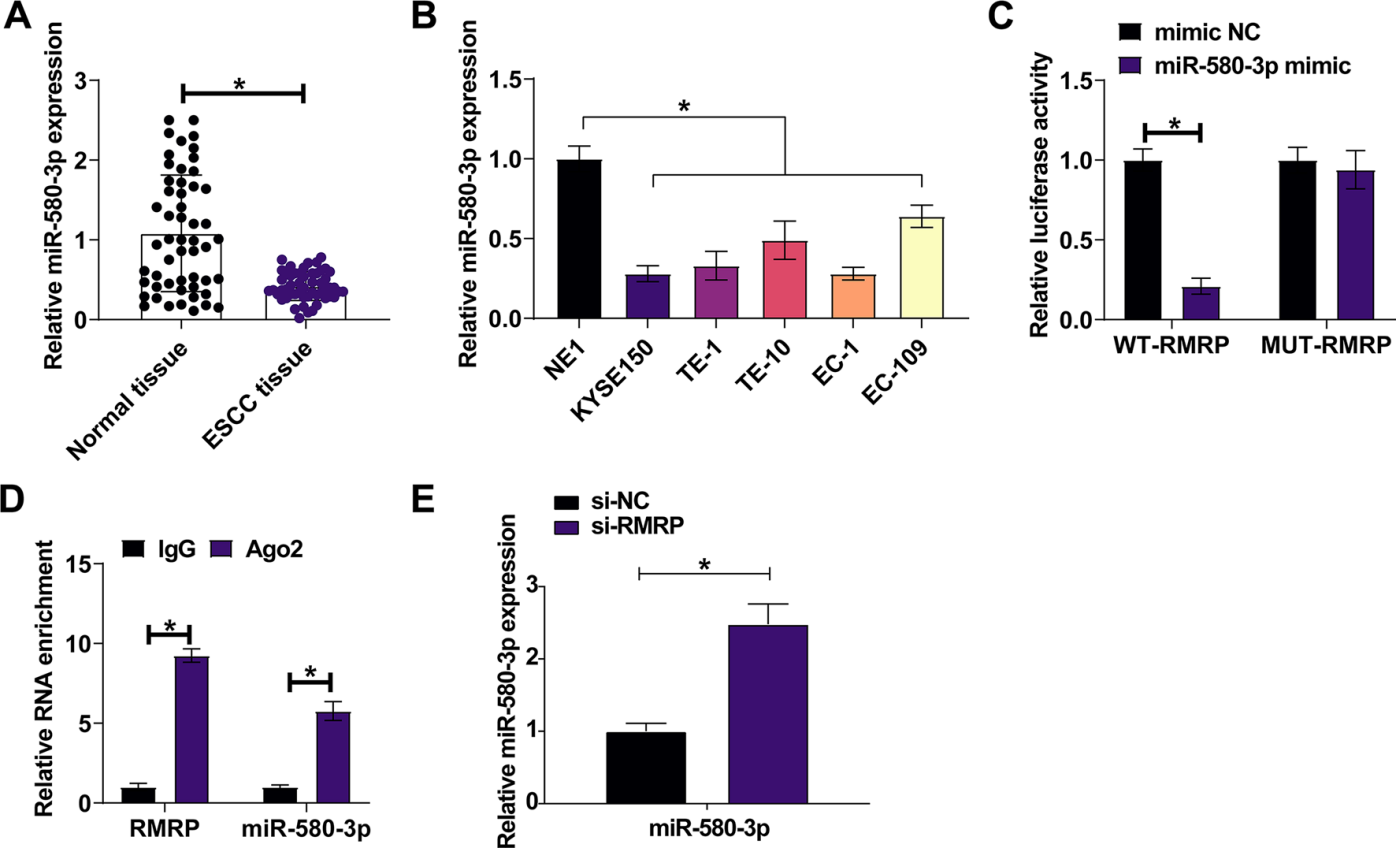
miR-580-3p is targeted by RMRP. **A** Quantitative PCR assessed miR-580-3p in patients’ tissues. **B** Quantitative PCR assessed miR-580-3p in ESCC cell lines and normal esophageal epithelial cells. **C**, **D** Dual luciferase reporter assay and RIP assay confirmed the targeting relationship between miR-580-3p and RMRP. **E** Quantitative PCR analyzed miR-580-3p after knocking down RMRP. Data were expressed as mean ± SD (N = 3). *P < 0.05

Bioinformatics website starbase predicted potential binding sites between RMRP and miR-580-3p. In the setting of ESCC, quantitative PCR assessed miR-580-3p expression and discovered its low expression pattern in ESCC tissues and cell lines compared with normal controls (Fig. 3A, B). Results of dual luciferase assay demonstrated that miR-580-3p mimic had the ability to reduce the luciferase activity of WT-RMRP (Fig. 3C). Moreover, RIP assay further confirmed that Ago2 was able to enrich miR-580-3p and RMRP in the immunoprecipitates (Fig. 3D). Concerning to miR-580-3p expression mediated by RMRP, quantitative PCR revealed that RMRP deficiency forced miR-580-3p expression in cells (Fig. 3E).

### RMRP promotes the malignant behavior of ESCC by adsorption of miR-580-3p

**Fig. 4:**
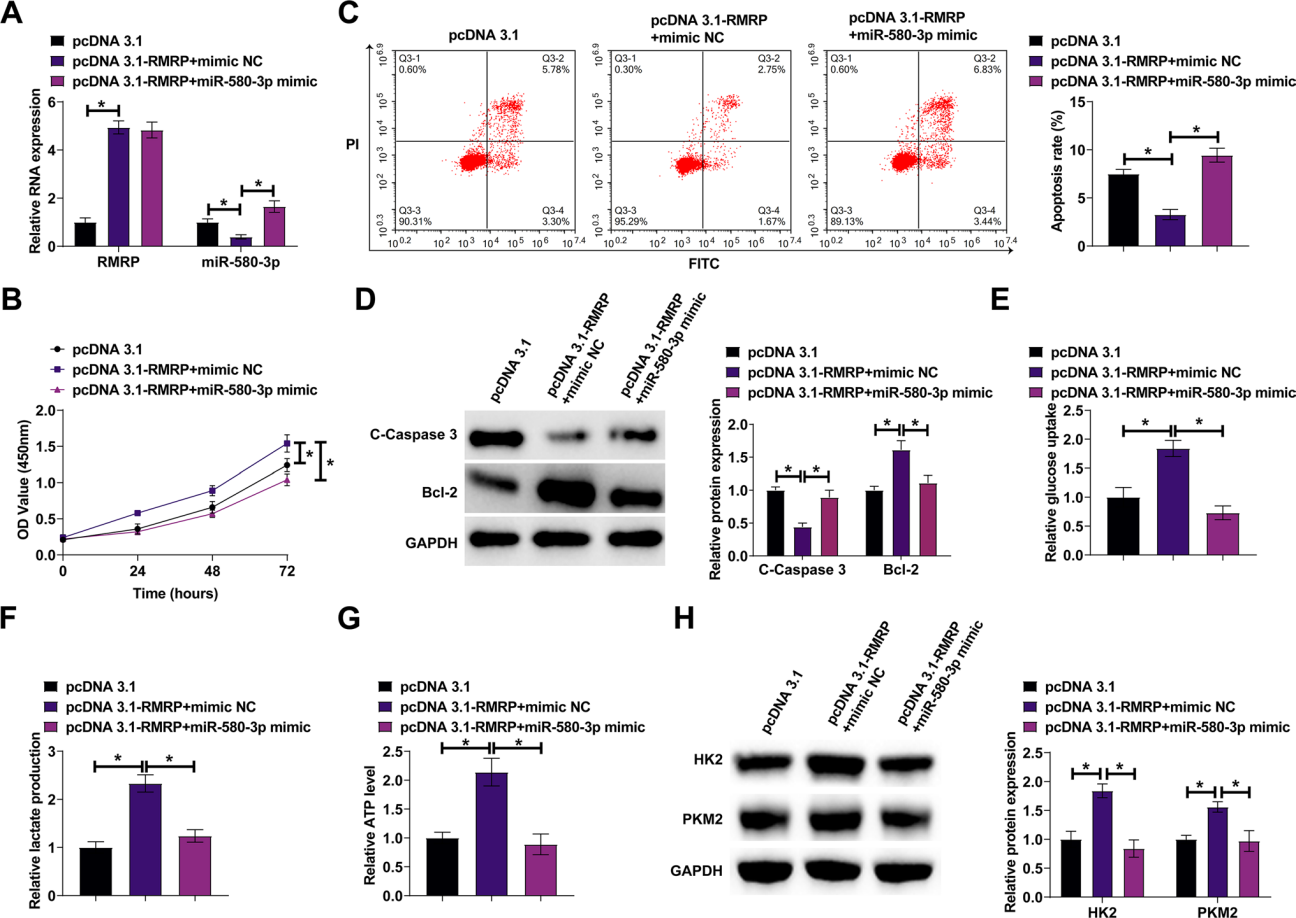
RMRP promotes ESCC by adsorption of miR-580-3p. pcDNA 3.1-RMRP and miR-580-3p mimic were co-transfected into KYSE150 cells. **A** Quantitative PCR assessed RMRP and miR-580-3p. **B** CCK-8 assay measured cell proliferation rate. **C** Flow cytometry detected cell apoptosis rate. **D** Western blot evaluated cleaved Caspase-3 and Bcl-2. **E**–**G** Commercial kits detected cellular glucose consumption, lactate production, and ATP levels. **H** Western blot evaluated HK2 and PKM2. Data were expressed as mean ± SD (N = 3). *P < 0.05

pcDNA 3.1-RMRP and miR-580-3p mimic were co-transfected into KYSE150 cells. pcDNA 3.1-RMRP forced RMRP and reduced miR-580-3p expressions, but miR-580-3p mimic could restore miR-580-3p expression (Fig. 4A). CCK-8 results illustrated that pcDNA 3.1-RMRP promoted cell proliferation rate, but miR-580-3p mimic blocked this phenomenon (Fig. 4B). Flow cytometry identified that pcDNA 3.1-RMRP led to the reduction of apoptosis rate, but miR-580-3p mimic re-activated cellular apoptosis (Fig. 4C). Western blot showed that pcDNA 3.1-RMRP increases the expression level of cleaved Caspase-3, and decreases the expression level of Bcl‑2, which were rescued by miR-580-3p mimic (Fig. 4D). Furthermore, pcDNA 3.1-RMRP increased glucose consumption, lactic acid production, and ATP productions and enhanced HK2 and PKM2 expressions, but this effect was suppressed after restoring miR-580-3p (Fig. 4E–H).

### miR-580-3p shares a targeting relationship with ATP13A3

**Fig. 5:**
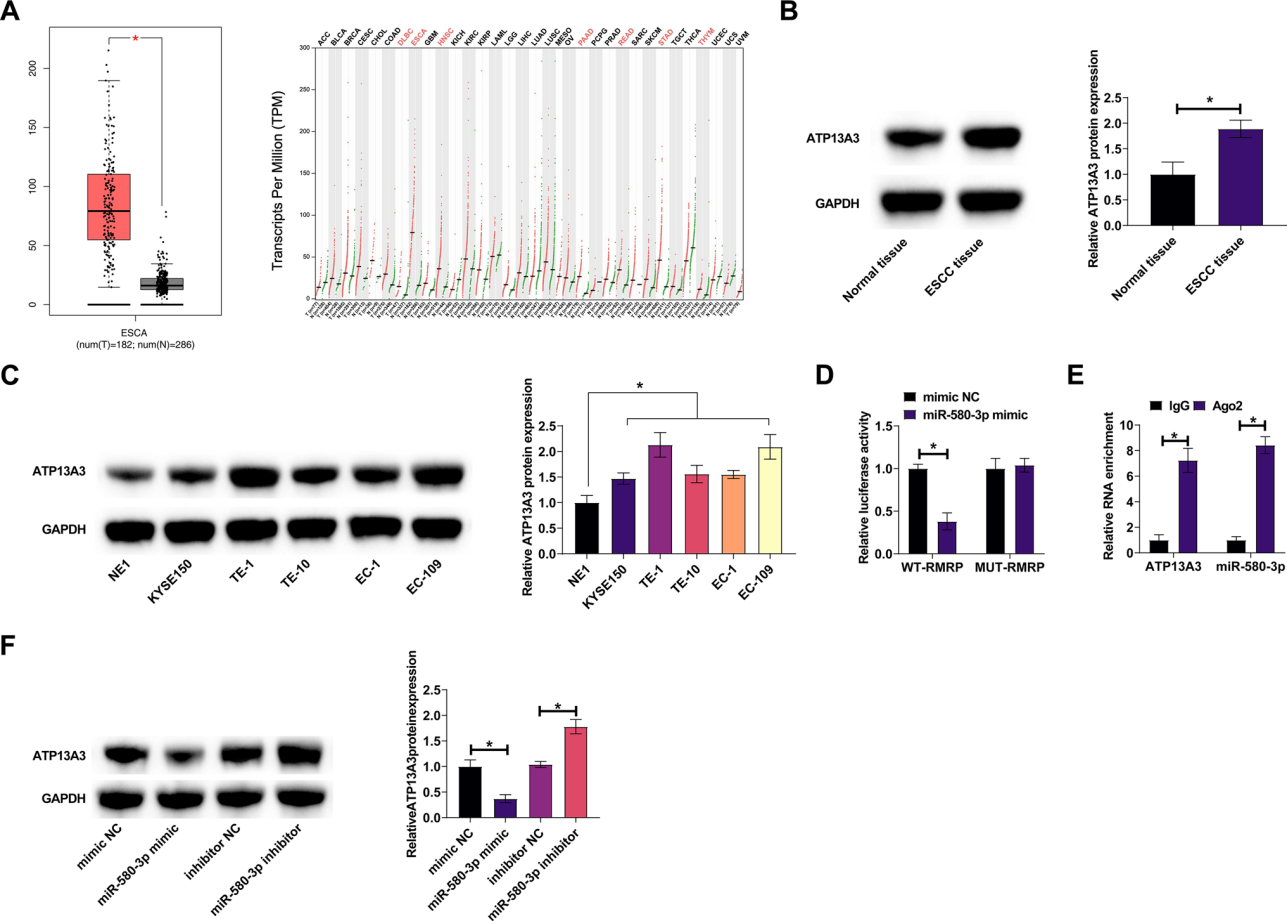
ATP13A3 is mediated by miR-580-3p. **A** GEPIA database analyzed ATP13A3 in ESCC patients. **B** Western blot assessed ATP13A3 in patients’ tissues. **C** Western blot assessed ATP13A3 in ESCC cell line and normal esophageal epithelial cells. **D**, **E** Dual luciferase reporter assay and RIP assay confirmed the targeting relationship between miR-580-3p and ATP13A3. **F** Western blot assessed ATP13A3 after regulating miR-580-3p. Data were expressed as mean ± SD (N = 3). *P < 0.05

Bioinformatics website predicted the potential binding sites of ATP13A3 and miR-580-3p. GEPIA data confirmed that ATP13A3 expression was rich in ECSS (Fig. 5A), and the same expression trend was observed in ESCC tissues and cell lines (Fig. 5B, C). Both dual luciferase assay and RIP assay confirmed the targeting relationship between ATP13A3 and miR-580-3p (Fig. 5D, E). Additionally, upregulating or downregulating miR-580-3p inhibited and promoted ATP13A3 expression, respectively (Fig. 5F).

### RMRP induces ESCC development by mediating ATP13A3

**Fig. 6:**
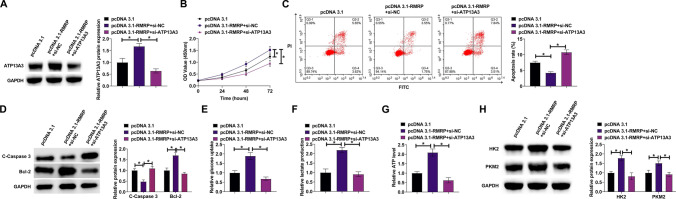
RMRP induces ESCC development by mediating ATP13A3. pcDNA 3.1-RMRP and si-ATP13A3 were co-transfected into KYSE150 cells. **A** Western blot assessed ATP13A3. **B** CCK-8 assay measured cell proliferation rate. **C** Flow cytometry detected cell apoptosis rate. **D** Western blot evaluated cleaved Caspase-3 and Bcl-2. **E**–**G** Commercial kits detected cellular glucose consumption, lactate production, and ATP levels. **H** Western blot evaluated HK2 and PKM2. Data were expressed as mean ± SD (N = 3). *P < 0.05

pcDNA 3.1-RMRP and si-ATP13A3 were co-transfected into KYSE150 cells. pcDNA 3.1-RMRP elevated ATP13A3 levels, but this effect was reversed by si-ATP13A3 (Fig. 6A). Functional rescue experiments mentioned that the pro-tumor effect of pcDNA 3.1-RMRP on proliferation, apoptosis, and glycolysis as above tested could be prevented after knocking down ATP13A3 (Fig. 6B–H).

## Discussion

About 90% of esophageal cancers are ESCC, which has a poor prognosis and high mortality [19]. It is s argued that cell proliferation requires energy, nutrients, and biosynthesis to replicate macromolecular components in cell passages. Glycolysis is primarily to maintain high levels of glycolic intermediates to support metabolic requirements of cell proliferation [20]. Unlike normal healthy cells, cancer cells are more metabolically active, proliferate at a higher rate, and are able to resist cell death pathways such as apoptosis [21]. Considering the significance of glycolysis during cancer cell proliferation and apoptosis, this work paid much attention to RMRP’s roles in regulating tumor glycolysis to interfere with ESCC progression.

As the findings indicated, RMRP exhibited high expression levels in ESCC and indicated a close association with larger tumor size and distal metastasis, as well as poor 5-year survival rates. In addition to that, cell experiments observed that silencing RMRP suppressed proliferation rates, elevated apoptosis rates, lowered glucose consumption, lactic acid production, and ATP levels, and inhibited HK2 and PKM2 protein expression, providing evidence that RMRP prevented proliferative and glycolytic activities and induced apoptosis during ESCC cell growth. Actually, RMRP is considered and identified as an emerging target in carcinogenesis and tumor therapy [22] and is a potent regulator of metabolic reprogramming in cancer [23]. Specifically, highly expressed RMRP has been studied in bladder cancer patients and is associated with tumor size, lymph node metastasis, and patients’ survival [24]. Furthermore, RMRP has been identified as an upregulated lncRNA in non-small cell lung cancer, and it acts mainly on tumor cells in part by forcing cell proliferation [25]. Notably, Chen et al. report RMRP expression abundance in colorectal cancer and this abundance is related to an unfavorable prognosis, and moreover, RMRP can facilitate tumor cell proliferation depending on p53 [26]. Glycolysis is the backbone of cancer cell metabolism, and cancer cells have evolved various mechanisms to enhance it [33]. Evidence swiftly accumulates of lncRNAs influencing glycolysis of cancer cells. In ESCC, researchers reported many lncRNAS have emerged as potent regulators of glycolysis, such as lncRNA G077640 [34], lncRNA-LET [35], lncRNA PTPRG-AS1 [34], et al. Concerning the correlation between RMRP and glycolysis, a report has illustrated that RMRP deficiency contributes to the reduction of glucose uptake, lactic acid, and ATP production in ovarian cancer cells resistant to paclitaxel [16]. In the setting of ESCC, an updated paper has verified the upregulation of RMRP in ESCC and further checked the anti-proliferation nature of depleted RMRP in ESCC cells [12]. All these papers, together with the support of this research, confirm the involvement of RMRP in tumor development by mediating malignant activities including glycolysis, proliferation, and apoptosis.

LncRNA can decoy miRNA to reduce regulatory effect on mRNA. This capability introduces additional complexity in miRNA-target interaction networks [27]. This study identified the downregulated miRNA miR-580-3p in ESCC to interact with RMRP. According to the research data, overexpressing RMRP further enabled ESCC cell proliferation, glycolysis, and anti-apoptosis. However, upregulating miR-580-3p led to the reversal of this promoting effect. It is addressed that miR-580-3p downregulation is shown in ovarian cancer, and suppressing miR-580-3p is contributed to cancer cell proliferation [28]. Moreover, miR-580-3p has been revealed to be downregulated in glioma, and enhancement of miR-580-3p induces a decline in cell proliferation [29]. Peng et al. confirm that miR-580-3p expression is maintained at low expression levels in melanoma and its high expression imposes suppressive effects on cell proliferation and anti-apoptosis [17]. As to mRNA regulated by miR-580-3p, this research selected ATP13A3 as a study interest. ATP13A3 is a P-type ATPase that is highly expressed in pancreatic cancer patients with unacceptable survival [30]. Lek et al*.* notice that ATP13A3 is overexpressed in head and neck squamous cell carcinoma [31], which is consistent with the present study findings. In ESCC cells, the study further analyzed ATP13A3-related functions and supported that depleting ATP13A3 suppressed RMRP-induced malignant proliferation, glycolysis, and anti-apoptosis.

To summarize, RMRP stimulates ESCC proliferative, glycolytic, and anti-apoptotic activities by sequestering miR-580-3p to mediate ATP13A3 expression. The main conclusion offers a lncRNA-targeting niche for managing tumor development by formulating metabolic reprogramming. However, whether RMRP can program other malignant phenotypes of ESCC cells still requires further analysis. In addition, the study only performed cell experiments, and animal experiments will conduct in future studies to validate these in vitro findings.

### Supplementary Information

**Additional file 1.**

**Additional file 2.**

## Data Availability

The datasets used and/or analyzed during the present study are available from the corresponding author on reasonable request.
